# Factors influencing attempted and completed suicide in postnatal women: A population-based study in Taiwan

**DOI:** 10.1038/srep25770

**Published:** 2016-05-12

**Authors:** Shu-Chuan Weng, Jung-Chen Chang, Ming-Kung Yeh, Shun-Mu Wang, Yi-Hua Chen

**Affiliations:** 1 School of Public Health, College of Public Health and Nutrition, Taipei Medical University, Taipei, Taiwan; 2 School of Nursing, College of Medicine, National Taiwan University, Taipei, Taiwan; 3 School of Pharmacy, Graduate Institute of Medical Science, National Defense Medical Center, Taipei, Taiwan; 4 Department of Public Health, China Medical University, Taichung, Taiwan; 5 Department of Health Services Administration, China Medical University, Taichung, Taiwan; 6 Department of Health Care Administration, Oriental Institute of Technology, New Taipei, Taiwan

## Abstract

The aims of study were to investigate risk factors associated with attempted and completed suicide. This nested case–control study was conducted using the medical and death data of nearly all pregnant women for the period 2002–2012 in Taiwan. A total of 139 cases of attempted suicide and 95 cases of completed suicide were identified; for each case, 10 controls were randomly selected and matched to the cases according to age and year of delivery. A conditional logistic regression model was used. The mean attempted and completed suicide rates were 9.91 and 6.86 per 100,000 women with live births, respectively. Never having married and postpartum depression also increased the risk of attempted suicide (OR = 2.06; 95% CI = 1.09–3.88 and OR = 2.51; 95% CI = 1.10–5.75, respectively) and completed suicide (OR = 20.27; 95% CI = 8.99–45.73 and OR = 21.72; 95% CI = 8.08–58.37, respectively). Other factors for attempted suicide included being widowed or divorced, and having a caesarean delivery or suicide history. Other factors for completed suicide included lower education level, low infant birth weight, and diagnosis of anxiety or mood disorder. These results suggest that people should appropriately assess potential risk factors and provide assistance for postnatal women to reduce the occurrence of suicide events.

Suicide is a serious concern worldwide^1^ and a critical contributor to maternal mortality^2^. The suicide mortality rate in postpartum women is lower than that in women of the same age who have not been pregnant^3^,^4^. Nevertheless, suicide is considered a serious problem in postnatal women because of its profound impacts on the risk to the woman’s life, the growth and development of the baby, the function of the family, and possibly the burden on the community as a whole^5^. Postpartum depression is a common complication of childbearing^6^ and has been identified as a major risk of suicide^7^. Suicidal behaviours include suicidal ideation as well as attempted and completed suicide^8^. According to a systematic literature review of data from both developed and developing countries, the prevalence of suicidal ideation and attempted suicide during the perinatal period is approximately 5%–14% and 0.125%–0.2%, respectively^4^. Among all pregnancy-associated deaths, 3%–7% are the result of suicide^9^.

Previous studies examining suicidal ideation and attempted suicide among perinatal women have used self-screening instruments, such as the Edinburgh Postnatal Depression Scale (EPDS) and Mini International Neuropsychiatric Interview (MINI)^10^,^11^, to evaluate suicidal ideation and attempted suicide. Various risk factors are associated with suicidality during the perinatal period. The known risk factors for attempted suicide in perinatal women include younger age, unmarried status, a lower education level, previous suicide attempts, intimate partner violence, alcohol or drug use, and psychiatric disorders such as anxiety and depression^10^,^12^,^13^,^14^. In addition, maternal and infant health status might affect suicidality^15^,^16^.

Only a few studies have evaluated the risk factors for completed suicide among perinatal women. Palladino *et al.*^2^ and Gold *et al.*^5^ have used the data of the National Violent Death Reporting System, combining data from death certificates, coroner or medical examiner reports, toxicology findings, and law enforcement reports to investigate pregnancy-associated homicide and suicide in 17 US states during 2003–2007. Palladino *et al.*^2^ analysed the victim characteristics in 94 cases of completed pregnancy-associated suicide and reported that intimate partner conflict appeared to contribute to 54.3% of the cases. Gold *et al.*^5^ examined 2083 female suicide victims and found that mental health, substance use, and intimate partner conflict were associated with pregnancy-associated suicide.

Previous reports on the factors influencing attempted and completed suicide among postnatal women have been insufficient. Although studies by Palladino *et al.*^2^ and Gold *et al.*^5^ have been strengthened by the use of multiple data sources, a lack of comprehensive information regarding healthcare utilisation limits understanding of women’s physical and mental health status before they take their lives. The use of a questionnaire to assess suicidality in many of previous studies may entail self-report bias, particularly considering the sensitivity of the topic, whereas examining healthcare utilisation records would be less prone to such biases and provide more robust results, especially for severe forms of suicide-related behaviour. Nearly no systematic examinations of suicide attempts that caused serious injuries requiring immediate medical treatment have been performed. Furthermore, few studies have simultaneously investigated the risk factors for both attempted and completed suicide, particularly over a study period longer than 10 years. Conducting such a study is imperative for suicide prevention and intervention.

In this study, we used the medical and death data of nearly all pregnant women in Taiwan for the period 2000–2012. Cases of attempted suicide undoubtedly share certain similar traits with those of completed suicide; however, there are clear differences in terms of the underlying pathology and risk factors for these behaviours^14^,^17^. No study has substantially compared attempted and completed suicide for postnatal women. Therefore, the aims of this study were to investigate sociodemographic variables (e.g. maternal marital status and education level), maternal psychiatric history (e.g. history of psychiatric disorders and suicide), and maternal and infant health conditions as potential risk factors for attempted and completed suicide within 1 year post partum. The rates of attempted and completed suicide for the decade from 2002–2012 were further examined. We hypothesised that poorer sociodemographic status (e.g. lower education and lower income), a history of more psychiatric disorders (e.g. depression), and adverse maternal and infant health conditions might be associated with higher risks of attempted and completed suicide.

## Materials and Methods

### Data Source

The data used in this nested case–control study were collected for the period 2000–2012 from the Health and Welfare Data Science Center (HWDC), Ministry of Health and Welfare, Taiwan, which is linked to the National Birth Registry, National Death Certification Registry, and National Health Insurance Research Database (NHIRD) by national identification numbers. In Taiwan, all birth and death information must be recorded in the Birth Registry and Death Certification Registry, and this information has been evaluated and determined to be valid and complete^18^,^19^,^20^. The Birth Registry contains information on various birth characteristics that was filed by hospitals after maternal delivery, including sex, birth weight, gestational age, whether the birth was single or multiple, birth order, and the physical condition of the mother and infant. We also examined data on the marital status and education level of residents at the end of each year from the Household Registration Database in Taiwan. The National Death Certification Registry includes data on sex, year of birth, and date and cause of death for all Taiwan residents^21^. The National Health Insurance program covers more than 99% of the Taiwan population, and the NHIRD contains diagnosis and reimbursement details including hospitalisation, outpatient, and emergency treatment records. The NHIRD accurately represents the medical utilisation of Taiwan residents^22^. According to HWDC regulations, cells that have a number of patients less than 2 are not permitted to appear in the table to avoid the possible identification of patients. For example, in the nonattempted suicide group in our study, less than 2 individuals had a history of suicide. Therefore, the exact number was unknown and unable to be found. This study was approved by the Taipei Medical University-Joint Institutional Review Board, Taiwan (No. 201407036).

### Identification of Cases and Controls

The National Birth Registry contains data on 2,312,084 cases for the period between 1 January 2000 and 31 December 2012. We excluded data involving stillbirths and women who had missing age data or were aged <18 or >50 years. Multiple births, few of which were present in the data, were excluded because they are associated with factors that may have distorted the results (e.g. more health problems, higher mortality risks, increased loads for maternal care). Because of the long study period (2000–2012), more than one birth by the same woman may have been included in the data. To ensure the independence of each observation and avoid excluding suicide cases that occurred after a subsequent childbirth, the final record was selected for women who had more than one delivery. A total of 1,521,107 women were eligible for inclusion in this study.

The study investigated attempted and completed suicide separately. To ensure that each woman’s medical history was traced 2 years before their delivery, cases of attempted and completed suicide between 2002 and 2012 were identified from data for 2000–2012. Specifically, the attempted suicide group comprised 139 cases of women with an emergency, outpatient, or hospitalisation diagnosis recorded in the NHIRD for ‘suicide and self-inflicted poisoning or injury’ (ICD-9: E950–E959) within 1 year following delivery between 2002 and 2012)^23^. These women were admitted to hospitals for a serious suicide attempt, which is distinct from nonsuicidal or nonserious self-harm. The completed suicide group comprised 95 cases extracted from the Death Certification Registry (ICD-9: 950–959 and 980–989 during 2002–2007; ICD-10: X60–X84, Y10–Y34, and Y87.0 during 2008–2012)^24^ occurring within 1 year following delivery. Control groups for the attempted and completed suicide groups were created by randomly selecting 10 controls with no attempted or completed suicide diagnoses, respectively, for each case and matching them by age and year of delivery according to the principles proposed by Rothman^25^ and Hennessy *et al.*^26^ The attempted and completed suicide control groups comprised 1,390 and 950 patients, respectively. All patients were followed through the claims and death registry records for 1 year after delivery or until 31 December 2012.

### Variable Descriptions and Definitions

The dependent variable was suicidal behaviour within 1 year following delivery for both the attempted and completed suicide groups. The independent variables were maternal and neonatal status, as obtained from the Birth Registry. Neonatal status comprised neonatal sex (male or female), birth weight (<2500 g or ≥ 2500 g), Apgar score (appearance, pulse, grimace, activity, and respiration at 1 and 5 minutes; <7, ≥ 7, or unknown), and congenital malformations (yes or no; e.g. heart defects, cleft lip or palate, and limb defects).

Maternal status comprised marital status at the end of the previous year (married, never married, widowed or divorced, or unknown), education level at the end of the previous year (<9 years, 9–12 years, >12 years, or unknown), delivery process (vaginal or caesarean delivery), use of a subsidy provided for qualified low-income NHI enrolees (yes or no), gestational diabetes mellitus (yes or no), gestational hypertension (yes or no), suicidal behaviour before the present delivery (ICD-9: E950.X–E959.X), pre-eclampsia or eclampsia during pregnancy (ICD-9: 642.4–642.7), and mental illness. The mental illnesses, diagnosed by a psychiatrist or physician, included anxiety (ICD-9: 300.X except 300.4) and mood disorder (ICD-9: 296.X and 300.4)^27^ 2 years before delivery and postpartum depressive disorder (ICD-9: 296.X and 300.4) before suicidal behaviour. The number of the cases with other psychiatric illnesses, such as bipolar disorder, schizophrenia, and substance use disorder, was too few to perform further analysis. Information on medical history was obtained from the NHIRD. To ensure the validity of diagnoses, anxiety and mood disorder were identified according to the respective ICD-9 codes at least three times within 2 years before delivery^22^. Gestational diabetes mellitus, hypertension, pre-eclampsia, and eclampsia were identified according to the presence of the corresponding ICD-9 codes at least once within 1 year before delivery. Postpartum depressive disorder was identified according to the corresponding ICD-9 codes used at least once following delivery. Women were considered to have a history of suicide if they had received a related ICD-9 code (ICD-9: E950–E959) at least once before delivery.

### Statistical Analysis

The crude rates of attempted and completed suicide during the first year post partum were calculated for each study year by dividing the numbers of suicide attempts and deaths, respectively, occurring in a calendar year among postnatal women by the corresponding live birth estimates and then multiplying the result by 100,000.

A chi-squared analysis was performed to estimate factors associated with each dependent variable (i.e. attempted and completed suicide). Results from a literature review and statistical analysis were both considered in model selection. Variables that were significantly associated with each dependent variable (p < 0.1) were included in conditional logistic regression models (stratified by age and year of delivery) for estimating odds ratios (ORs) and 95% confidence intervals (CIs). The delivery method was significantly associated with attempted suicide, and pre-eclampsia or eclampsia was significantly associated with completed suicide. Both of these variables were crucial risk factors for maternal health^28^,^29^. Therefore, we retained these two variables in both the attempted and completed suicide models.

The significance level for all statistical analyses was *p* < 0.05. All data were analysed using SAS version 9.3 (SAS, Cary, NC, USA).

## Results

**Figure 1:**
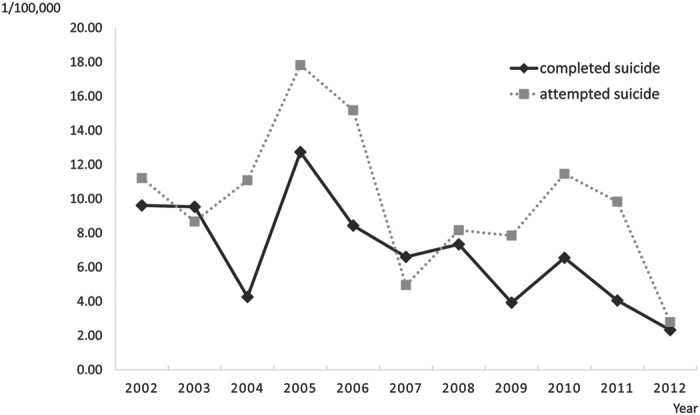
The attempted and complete suicide rates during one year postpartum from 2002 to 2012.

In total, 95 cases of completed suicide and 139 cases of attempted suicide were identified in postpartum gravidas. Sixty-six (72.53%) of the women who completed suicide were aged 25–34 years and 10 (10.99%) were aged 18–24 years; 83 (62.41%) of the women who attempted suicide were aged 25–34 years and 29 (21.8%) were aged 18–24 years. Figure 1 shows the trends of postnatal attempted and completed suicide between 2002 and 2012. The mean postnatal suicide mortality and attempted suicide rates were 6.85 and 9.91 per 100,000 live births, respectively. The trends of the rates were decreasing but exhibited two increases during 2005–2010. The highest rates occurred in 2005 and the lowest rates occurred in 2012, probably because of the short follow-up period among cases in 2012.

The overall characteristics of the patients in the attempted suicide group are presented in Table 1. We observed that the women who attempted suicide were significantly more likely than those who did not attempt suicide (p < 0.05) to have a lower education level (20.86% vs. 11.51%), no legal spouse at the end of the year before delivery (33.09% vs. 14.02%), received a caesarean delivery (53.24% vs. 29.71%), a child with a low birth weight (12.23% vs. 5.32%), a history of suicide before delivery (5.76% vs. <0.01%), a diagnosis of anxiety (3.6% vs. 0.86%) or mood disorder (10.79% vs. 0.43%) within 2 years before delivery, and a diagnosis with postpartum depressive disorder (25.9% vs. 1.08%). After adjustment for possible confounding variables, the risk of attempted suicide remained significantly associated with never having married (OR = 2.06; 95% CI = 1.09–3.88) or being widowed or divorced (OR = 5.06; 95% CI = 2.15–11.93) at the end of year before delivery, receiving a caesarean delivery (OR = 2.38; 95% CI = 1.56–3.62), a history of suicide before delivery (OR = 32.34; 95% CI = 3.52–297.11), and diagnosis with postpartum depressive disorder (OR = 20.27; 95% CI = 8.99–45.73). Neither birthing a female (p = 0.07) or low-birth-weight infant (p = 0.06) nor having maternal mood disorder 2 years before delivery (p = 0.05) were significantly associate with attempted suicide in the current models.

The overall characteristics of the patients in the completed suicide group are presented in Table 2. The women who completed suicide during the postpartum period were significantly more likely than those who did not complete suicide (p < 0.05) to have a lower education level (25.26% vs. 11.79%), no legal spouse at the end of previous year before delivery (27.36% vs. 10.21%), a child with a low birth weight (16.84% vs. 5.05%), a diagnosis of anxiety (12.63% vs. 1.16%) or mood disorder (17.89% vs. 0.95%) within 2 years before delivery, and a diagnosis of pre-eclampsia or eclampsia (5.26% vs. 1.37%) or postpartum depressive disorder (30.53% vs. 1.37%). None of the women who completed suicide had a history of suicide before delivery during the study period. After adjustment for possible confounding variables, the risk of completed suicide remained significantly associated with never having married (OR = 2.51; 95% CI = 1.10–5.75), having a low-birth-weight infant (OR = 2.89; 95% CI = 1.30–6.40), diagnosis with anxiety (OR = 10.71; 95% CI = 2.40–47.75) or mood disorder (OR = 5.82; 95% CI = 1.85–18.38) within 2 years before delivery, and diagnosis with postpartum depressive disorder following delivery (OR = 21.72; 95% CI = 8.08–58.37). Having received more than 12 years of education was independently associated with a reduced risk of completed suicide (OR = 0.17; 95% CI = 0.07–0.41).

We excluded all women who gave birth in 2012 because of the shorter follow-up period and reanalysed the data. We observed consistent results; therefore, the findings for all the examined women (including those who gave birth in 2012) were used in this study to improve its statistical power.

**Figure 2:**
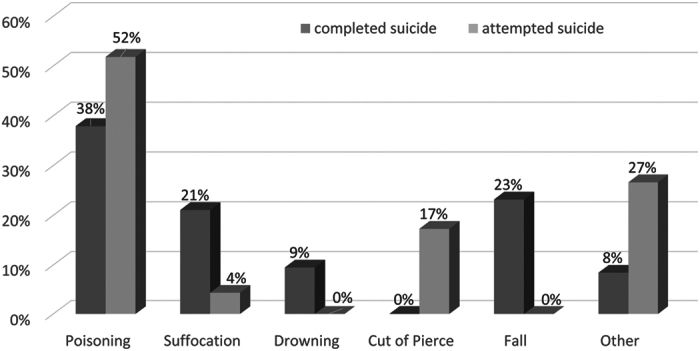
Distributions of various suicidal methods in women with attempted (n = 139) and complete suicide (n = 95).

The differences in suicide method between the attempted and completed suicide groups are illustrated in Fig. 2. Poisoning was the most common method in both groups. Suffocation, drowning, and falling are generally fatal; accordingly, these methods were more common among the women who completed suicide than among those who attempted suicide. The women who completed suicide were less likely to use methods such as cutting and piercing.

## Discussion

This study sought to investigate the risk factors associated with attempted and completed suicide, and to evaluate the rates of attempted and completed suicide among women within 1 year post partum. In this study, the mean attempted and completed suicide rates within 1 year following delivery in Taiwan during 2002–2012 were 9.91 and 6.86 per 100,000 women who had live births, respectively. By contrast, the mean suicide mortality rate of women aged 25–45 years during 2002–2012 was 12.1 per 100,000 in Taiwan^30^. The global age-standardised suicide rate in 2012 was 8.0 per 100,000 females^1^.

In this study using national data highly representative of the Taiwan population, the suicide mortality rate of postnatal women was lower than that of women of the same age in the general population, which is consistent with the results of other studies^4^,^31^. However, for postnatal women, the suicide mortality rate in our study is lower than that reported by Gissler *et al.*^32^ in Finland for the period 1987–2000^32^, but higher than that reported by Palladino *et al.*^2^ in 17 US states for 2003–2007^2^, which included both mortality occurring during pregnancy and post partum. Further examination of the current data revealed that the suicide mortality rate among postnatal women peaked in 2005, which is similar to the overall trend of suicide mortality among females in Taiwan. The suicide mortality rate declined considerably after the Taiwan Suicide Prevention Center was established in 2005. In addition, the rates of attempted and completed suicide may vary considerably from year to year because of the small numbers of events among postnatal women in each year (as indicated by the totals of 139 and 95 cases, respectively, for the entire period of 2002 to 2012).

Although attempted suicide has been reported to occur more frequently than completed suicide, the rates of attempted and completed suicide in our study did not differ substantially. In fact, previous studies have reported attempted suicide rates determined using questionnaires to be 1–5%^13^,^33^, which is far higher than that observed in our study, possibly because we identified cases of attempted suicide in which self-harm behaviour was sufficiently severe to require medical treatment. Cases of attempted suicide involving only slight injuries were not included in this study.

Poor mental health, low socioeconomic status, and nonmarried status are reported risk factors for suicide in the general population^34^,^35^. In addition to identifying these risk factors in the current study, we found that postnatal women who have a caesarean delivery, have a history of suicide, are unmarried, or have postpartum depressive disorder are also at an increased risk of attempted suicide. Previous studies have considered a history of suicide, unmarried status, and postpartum depressive disorder as crucial risk factors for attempted suicide during perinatal periods^7^,^14^, which is consistent with our results. Gandhi *et al.*^36^ observed that women who attempted suicide during pregnancy more frequently underwent caesarean delivery^36^. However, Schiff and Grossman^37^, investigating women in Washington state during 1987–2001, did not find any association between caesarean delivery and attempted suicide^37^. The caesarean delivery rate in Taiwan was more than 30% in the past decade, with most caesarean deliveries performed upon maternal request^38^. In our study, postnatal women who underwent caesarean delivery experienced an increased risk of attempted suicide. Indeed, previous studies have determined that women who electively underwent a caesarean delivery were more likely to display introversive and emotionally unstable personalities^39^ as well as lower self-efficacy^40^. Women with these traits have been observed to have increased risks of suicidal behaviour^41^,^42^.

Postnatal women with a low-birth-weight infant, unmarried status, lower education level, and a history of depression, anxiety, and postpartum depressive disorder have an increased risk of completed suicide. In previous studies, having unmarried status, a low education level, a history of depression and anxiety, and postpartum depressive disorder were similarly considered substantial risk factors for suicidality^2^,^10^,^43^,^44^,^45^. Low birth weight can indicate that an infant is unwell. Moreover, adverse infant health status increases the risk of suicidal behaviour in the mother^46^.

The results of this study regarding postpartum women are consistent with the finding of Czeizel^14^ that the characteristics of people who complete suicide and attempt suicide are different^14^. The WHO (2014) reported that a prior attempted suicide is the largest risk factor for suicide in the general population^1^. However, in the current study, a prior attempted suicide was not associated with the risk of completed suicide in postnatal women, and no postnatal women who completed suicide were diagnosed with self-harm during the study period. The women who completed suicide used methods that result in death more directly and reliably than other methods. Younes *et al.*^47^ discovered that a history of attempted suicide is significantly less common among people who completed suicide than among those who attempted suicide^47^. In addition, the current study supported the finding of previous studies that depression and anxiety are highly associated with completed suicide^16^,^43^. However, for attempted suicide, we identified depression, but not anxiety, to be a marginally significant factor. Low infant birth weight was significantly associated with completed suicide and marginally associated with attempted suicide. In addition, Candance *et al.* (2011) determined that low birth weight was associated with intentional poisoning in California^48^. Furthermore, Schiff and Grossman^37^ found that low birth weight was associated with attempted suicide, but the association was nonsignificant after adjustment for main factors^37^, which is consistent with our results. In addition, we observed that women who underwent a caesarean delivery had a significantly increased risk of attempted suicide but not of completed suicide. Moreover, postpartum depressive disorder was a crucial risk factor for attempted and completed suicide. Compared with women who were not diagnosed with postpartum depressive disorder, those with postpartum depressive disorder had a more than 20-fold greater risk of both attempted and completed suicide.

The most common method of attempted and completed suicide was poisoning, which is consistent with the findings of other studies^5^,^37^. Firearm ownership in Taiwan is heavily restricted, and no women committed suicide by using a firearm. Lethal suicide methods such as hanging increase the rate of suicide completion^49^; self-poisoning and self-injury have lower fatality rates^49^,^50^.

In the past few years, more attention has been paid to preventing suicidal behaviour by perinatal women. More appropriate methods are required to detect perinatal women with a risk of attempted and completed suicide. In this study, we determined that the potential risk factors associated with attempted and completed suicide among postnatal women varied. A history of suicide has been identified as a crucial risk factor for completed suicide in previous studies^51^,^52^. This finding does not apply to the postnatal women in our study. The risk of suicidal behaviour might be underestimated in postnatal women without a history of suicide. Therefore, women with various risk factors for suicide, such as a lower education level, unmarried status, and history of mood disorders, must be given more attention. Postnatal women who have a tendency of suicidal behaviour must have their access to violent means of suicide restricted, including waterside areas, high-rise buildings, and materials that can be used for hanging. Moreover, the family members of postnatal women must care for and monitor them, particularly for those diagnosed with postnatal depressive disorder. Postnatal women frequently seek healthcare services for the infant and themselves during the initial few months following delivery. Medical staff must appropriately assess potential risk factors for suicide and provide prompt assistance for postnatal women to reduce the occurrence of suicide events. Using an instrument (e.g. EPDS) for the early screening and detection of depressive symptoms and suicide ideation or attempts, with treatment such as antidepressants or dialectic behaviour therapy implemented promptly to reduce emotional disturbances and self-harm, is paramount for ensuring the health of postnatal women^53^.

A strength of this study was the use of long-term population-based data, which have been used infrequently in similar previous studies. Moreover, we collected several crucial variables associated with suicidal behaviour such as marital status, education level, economic status, and health status (including mental disorders and chronic and maternity-related diseases). In addition, the use of claims data to assess attempted suicide renders the results more robust and less prone to self-report bias.

This study has a few limitations. First, the sample of attempted suicide cases was limited to those who received medical treatment for suicidal behaviour. Women who attempted suicide but were prevented or sustained only minor injuries not requiring professional medical attention were not included. This study focused on severe injury and death caused by suicidal behaviour in postnatal women, understanding of which is insufficient. The low event rates of suicide contributed to a smaller sample size, which limited our ability to conduct further stratification. Second, our study was unable to investigate some effects such as the use of prescription medications or the phenotypic characterisation of psychiatric disorders, because of the use of claims and registry databases. Previous studies have determined that the rates of using antidepressant among pregnant women were extremely low, at 5% in a Canadian sample and 14% in a US sample^54^. Among women who used antidepressants before pregnancy, almost 60% stopped taking them in the first trimester out of concern about possibly increased teratologic risks^55^. However, the adverse effects of a relapse of their illness (depression, anxiety, or even the risk of suicide) may be more harmful to mother and child^55^. Other risk factors associated with suicidal behaviour for women in general, such as intimate partner violence^56^, major life events^57^, and physical or sexual abuse^58^, were also unavailable in our data sources. Among these factors, intimate partner violence was most discussed in perinatal women. Furthermore, one study found that smoking seemed to explain suicidality risk more effectively than did other variables in the general population^59^. However, smoking behaviour among perinatal women in Taiwan is low^60^ and was unavailable in this claims data.

Third, the education level was determined according to the registration data of domestic schools. Some records may have been incorrectly entered, and education level could not be confirmed for those who studied overseas. Although younger women might not have completed their education yet, any underestimation of education levels would likely be comparable between the cases and controls because the groups were age-matched. Finally, attempted and completed suicide might have been underascertained for reasons such as the social stigma associated with suicide and false diagnosis suicide ICD-9 codes for life insurance and other purposes. Women may be reluctant to report suicide attempts, particularly during the postpartum period, because of the possible removal of their child from their care if the safety of the child is seen as being at risk. Chang *et al.* reported that suicide rates in Taiwan may be underestimated by more than 30% because some suicides are ‘hidden’ among deaths attributed to other causes^61^.

Suicidal behaviour during the postnatal period must be prevented more effectively. The current study revealed that unmarried status and postpartum depressive disorder are associated with an increased risk of both attempted and completed suicide. However, the other risk factors varied between attempted and completed suicide. More clearly defining risk factors for suicide would facilitate identifying pregnant women with a higher risk of suicide and providing intervention promptly. Family members and medical staff can be more efficient in examining the risk of suicidal behaviour in postnatal women on the basis of our current results. Future research can focus on other suicide risk factors for women with postpartum depression, such as those related to social support system, specific stressors, and breastfeeding, by using a prospective study design.

## Additional Information

**How to cite this article**: Weng, S.-C. *et al.* Factors influencing attempted and completed suicide in postnatal women: A population-based study in Taiwan. *Sci. Rep.* **6**, 25770; doi: 10.1038/srep25770 (2016).

## Figures and Tables

**Table 1:** Risk of attempted suicide within 1 year post partum stratified by perinatal and other associated factors (n = 1529).

	Attempted suicide n = 139 n (%)	No attempted suicide n = 1390 n (%)	Chi- squared	*P* value	OR^a.^	95% CI	*P* value
Education			14.558	0.002			
<9 years (ref.)	29(20.86)	160(11.51)			1		
9–12 years	48(34.53)	417(30.00)			0.94	0.50–1.78	0.854
>12 years	24(17.27)	357(25.68)			0.57	0.27–1.21	0.14
Unknown	38(27.34)	456(32.81)			0.67	0.07–6.66	0.736
Low income			2.602	0.100			
No (ref.)	136(97.84)	1379(99.21)			1		
Yes	3(2.16)	11(0.79)			1.46	0.19–11.39	0.719
Marital Status			59.246	<0.0001			
Married (ref.)	56(40.29)	752(54.1)			1		
Never married	27(19.42)	162(11.65)			2.06	1.09–3.88	0.025
Widowed/divorced	19(13.67)	33(2.37)			5.06	2.15–11.93	0.000
Unknown	37(26.62)	443(31.87)			1.22	0.12–12.13	0.864
Delivery method			32.219	<0.0001			
Vaginal delivery (ref.)	65(46.76)	977()70.29			1		
Caesarean delivery	74(53.24)	413(29.71)			2.38	1.56–3.62	<0.0001
Infant sex			3.297	0.069			
Male (ref.)	64(46.04)	752(54.1)			1		
Female	75(53.96)	638(45.9)			1.46	0.97–2.20	0.073
Birth weight			10.768	0.001			
≥ 2500 g (ref.)	122(87.77)	1316(94.68)			1		
<2500 g	17(12.23)	74(5.32)			1.91	0.97–3.75	0.060
Pre-eclampsia/eclampsia			1.268	0.260			
No (ref.)	135(97.12)	1368(98.42)			1		
Yes	4(2.88)	22(1.58)			0.94	0.21–4.25	0.938
Suicide history^b.^			–	–			
No (ref.)	131(94.24)	–			1		
Yes	8(5.76)	–			32.34	3.52–297.11	0.002
Anxiety			8.590	0.003			
No (ref.)	134(96.4)	1378(99.14)			1		
Yes	5(3.6)	12(0.86)			1.48	0.25–8.92	0.670
Mood disorder			100.118	<0.0001			
No (ref.)	124(89.21)	1384(99.57)			1		
Yes	15(10.79)	6(0.43)			4.12	0.99–17.14	0.052
Postpartum depressive disorder			241.436	<0.0001			
No (ref.)	103(74.1)	1375(98.92)			1		
Yes	36(25.9)	15(1.08)			20.27	8.99–45.73	<0.0001

^a^The OR (odds ratio) was calculated by performing conditional logistic regression conditioned on the maternal age and year of delivery. The results were adjusted for the other variables listed in the table.

^b^The number of women who had a suicide history before delivery in the control group was less than 2; therefore, it is not presented in the table.

**Table 2:** Risk of completed suicide within 1 year post partum stratified by perinatal and other associated factors (n = 1045).

	Completed suicide		Chi- square	*P* value	OR^a.^	95% CI	*P* value
	n = 95 n (%)	No completed suicide n = 950 n (%)					
Education			21.771	<0.0001			
<9 years (ref.)	24(25.26)	112(11.79)			1		
9–12 years	35(36.84)	276(29.05)			0.52	0.25–1.07	0.078
> 12 years	17(17.89)	327(34.42)			0.17	0.07–0.41	<0.0001
Unknown	19(20.0)	235(24.74)			0.97	0.14–6.55	0.973
Marital status			26.684	<0.0001			
Married (ref.)	52(54.74)	623(65.58)			1		
Never married	17(17.89)	73(7.68)			2.51	1.10–5.75	0.029
Widowed/divorced	9 (9.47)	24(2.53)			3.43	0.99–11.87	0.052
Unknown	17(17.89)	230(24.21)			0.31	0.04–2.26	0.249
Delivery method			0.542	0.462			
Vaginal delivery (ref.)	58(61.05)	616(64.84)			1		
Caesarean delivery	37(38.95)	334(35.16)			0.69	0.39–1.23	0.204
Infant sex			3.190	0.074			
Male (ref.)	43(45.26)	521(54.84)			1		
Female	52(54.74)	429(45.16)			1.57	0.92–2.69	0.099
Low birth weight			20.879	<0.0001			
≥2500 g (ref.)	79(83.16)	902(94.95)			1		
<2500 g	16(16.84)	48(5.05)			2.89	1.30–6.39	0.009
Pre-eclampsia/eclampsia			7.7389	0.005			
No (ref.)	90(94.74)	937(98.63)			1		
Yes	5(5.26)	13(1.37)			2.06	0.46–9.20	0.342
Anxiety			52.819	<0.0001			
No (ref.)	83(87.37)	939(98.84)			1		
Yes	12(12.63)	11(1.16)			10.71	2.40–47.75	0.002
Mood disorder			102.2399	<0.0001			
No (ref.)	78(82.11)	941(99.05)			1		
Yes	17(17.89)	9(0.95)			5.82	1.85–18.38	0.003
Postpartum depressive disorder			190.338	<0.0001			
No (ref.)	66(69.47)	937(98.63)			1		
Yes	29(30.53)	13(1.37)			21.72	8.08–58.37	<0.0001

The OR (odds ratio) was calculated by performing conditional logistic regression conditioned on the maternal age and year of delivery. The results were adjusted for the other variables listed in the table.
